# Histone modification enhances the effectiveness of IL-13 receptor targeted immunotoxin in murine models of human pancreatic cancer

**DOI:** 10.1186/1479-5876-9-37

**Published:** 2011-04-08

**Authors:** Toshio Fujisawa, Bharat H Joshi, Raj K Puri

**Affiliations:** 1 Tumor Vaccines and Biotechnology Branch, Division of Cellular and Gene Therapies, Center for Biologics Evaluation and Research, Food and Drug Administration, Bethesda, MD, USA

## Abstract

**Background:**

Interleukin-13 Receptor α2 (IL-13Rα2) is a tumor-associated antigen and target for cancer therapy. Since IL-13Rα2 is heterogeneously overexpressed in a variety of human cancers, it would be highly desirable to uniformly upregulate IL-13Rα2 expression in tumors for optimal targeting.

**Methods:**

We examined epigenetic regulation of *IL-13Rα2 *in a murine model of human pancreatic cancer by Bisulfite-PCR, sequencing for DNA methylation and chromatin immunoprecipitation for histone modification. Reverse transcription-PCR was performed for examining changes in IL-13Rα2 mRNA expression after treatment with histone deacetylase (HDAC) and c-jun inhibitors. *In vitro *cytotoxicity assays and *in vivo *testing in animal tumor models were performed to determine whether HDAC inhibitors could enhance anti-tumor effects of IL-13-PE in pancreatic cancer. Mice harboring subcutaneous tumors were treated with HDAC inhibitors systemically and IL-13-PE intratumorally.

**Results:**

We found that CpG sites in *IL-13Rα2 *promoter region were not methylated in all pancreatic cancer cell lines studied including IL-13Rα2-positive and IL-13Rα2-negative cell lines and normal cells. On the other hand, histones at IL-13Rα2 promoter region were highly-acetylated in IL-13Rα2-positive but much less in receptor-negative pancreatic cancer cell lines. When cells were treated with HDAC inhibitors, not only histone acetylation but also IL-13Rα2 expression was dramatically enhanced in receptor-negative pancreatic cancer cells. In contrast, HDAC inhibition did not increase IL-13Rα2 in normal cell lines. In addition, c-jun in IL-13Rα2-positive cells was expressed at higher level than in negative cells. Two types of c-jun inhibitors prevented increase of IL-13Rα2 by HDAC inhibitors. HDAC inhibitors dramatically sensitized cancer cells to immunotoxin in the cytotoxicity assay *in vitro *and increased IL-13Rα2 in the tumors subcutaneously implanted in the immunodeficient animals but not in normal mice tissues. Combination therapy with HDAC inhibitors and immunotoxin synergistically inhibited growth of not only IL-13Rα2-positive but also IL-13Rα2-negative tumors.

**Conclusions:**

We have identified a novel function of histone modification in the regulation of IL-13Rα2 in pancreatic cancer cell lines *in vitro *and *in vivo*. HDAC inhibition provides a novel opportunity in designing combinatorial therapeutic approaches not only in combination with IL-13-PE but with other immunotoxins for therapy of pancreatic cancer and other cancers.

## Introduction

Interleukin-13 Receptor α2 (IL-13Rα2) is a high affinity receptor for the Th2 derived cytokine IL-13 and a known cancer testis antigen [1,2]. IL-13Rα2 is over expressed in a variety of human cancers including malignant glioma, head and neck cancer, Kaposi's sarcoma, renal cell carcinoma, and ovarian carcinoma [3-7]. We have demonstrated previously that IL-13Rα2 can be effectively targeted by a recombinant immunotoxin, consisting of IL-13 and truncated *pseudomonas *exotoxin (IL-13-PE) [8-11]. IL-13-PE is highly cytotoxic to tumor cells *in vitro *and *in vivo *that express high levels of IL-13Rα2 [12]. Several phase I and II clinical trials, and one phase III clinical trial, evaluating the safety, tolerability, and efficacy of this agent have been completed in patients with recurrent glioblastoma multiforme [13,14]. Most recently, we have demonstrated expression of IL-13Rα2 in human pancreatic ductal adenocarcinoma [15]. Seventy-one percent of pancreatic tumors overexpressed IL-13Rα2 chain. Pancreatic tumors were also successfully targeted by IL-13-PE in an animal model of human cancer [15,16]. Thus, IL-13Rα2 is currently being assessed as a cancer therapy in a variety of preclinical and clinical trials [4,17,18]

The significance of IL-13Rα2 expression in cancer is not known and the mechanism of its upregulation is still not clear. Epigenetic mechanisms such as DNA methylation and histone modification are known to be involved in many disease pathogenesis including cancer [19]. DNA methylation occurs on cytosines that are followed by guanines (CpG dinucleotides) and is usually associated with gene silencing [20]. Histones are modified at several different amino acid residues and with many different modifications including methylation, acetylation, phosphorylation and ubiquitination. Some lysine residues can either be methylated or acetylated, and there are three different possibilities for each methylated site [21]. Histone modification can be transiently altered by the cell environment [22]. Mainly, gene expression is activated by histone acetylation and decreased by methylation. Histone acetylation induced by histone acetyltransferase (HAT) is associated with gene transcription, while histone hypoacetylation induced by histone deacetylase (HDAC) is associated with gene silencing [23].

HDAC inhibition results in increased acetylation in histones and causes over expression of some genes. HDAC inhibitors are grouped into various classes based on their structures [24]. Trichostatin A (TSA), suberoylanilide hydroxamic acid (SAHA), and sodium butyrate (NaB) are commonly studied HDAC inhibitors. These inhibitors induce cell growth arrest and apoptosis in a broad spectrum of transformed cells [25]. Because of these characteristics, HDAC inhibitors are being tested in the clinic for cancer therapy. Two HDAC inhibitors, SAHA and Romidepsin, are licensed by FDA for the treatment of cutaneous T-cell lymphoma [26].

In the present study, we have examined the epigenetic regulation of the *IL-13Rα2 *gene in pancreatic cancer cell lines and investigated whether the *IL-13Rα2 *gene can be modulated by epigenetic mechanisms. We have also examined the effect of HDAC inhibitors on IL-13Rα2 expression. We demonstrate for the first time that three different HDAC inhibitors dramatically upregulate IL-13Rα2 in pancreatic cancer cell lines expressing no or low levels of IL-13Rα2. These inhibitors also modestly upregulated IL-13Rα2 in cells expressing higher levels of IL-13Rα2. More importantly, HDAC inhibitors sensitized pancreatic tumor cells to IL-13-PE and mediated enhanced sensitivity even though these cells did not naturally express IL-13Rα2. A combination therapy of HDAC inhibitors and IL-13-PE demonstrated a pronounced anti-tumor effect in human tumor bearing immunodeficient mice indicating a synergistic impact on tumor response. Thus, a novel combination of HDAC inhibitors and IL-13-PE may have a prominent role in pancreatic cancer or other cancer therapies in the clinic.

## Materials and methods

### Cell culture and reagents

Pancreatic cancer cell lines and human umbilical vein endothelial cell line (HUVEC) were obtained from the American Type Culture Collection (Manassas, VA). Human normal gingival fibroblasts (HGF) was obtained from Sciencell (San Diego, CA) and human pancreatic ductal epithelial cells (HPE) from Cell Systems (Kirkland, WA). Renal cell carcinoma (PM-RCC) cell line was developed in our laboratory [4]. Recombinant IL-13-PE was produced and purified in our laboratory [9,11,27]. Trichostatin A (TSA), sodium butyrate (NaB) and SP600125 were purchased from Sigma-Aldrich (St. Louis, MO). SR11302 was purchased from Tocris Bioscience (Ellisville, MO). Suberoylanilide Hydroxamic Acid (SAHA) was purchased from Selleck (Houston, TX).

### Reverse transcription-PCR

Quantitative reverse transcription-PCR (qRT-PCR) and RT-PCR were performed as described previously [28,29] using a SYBR 1 reagent kit (Bio-Rad, Hercules, CA). Mouse IL-13Rα2 and β-actin primers were purchased from QIAGEN (Valencia, CA). Gene expression was normalized to β-actin before the fold change in gene expression was determined.

### Chromatin immunoprecipitation (ChIP) assays

ChIP assays were performed using a ChIP assay kit (Millipore, Billerica, MA). To cross-link DNA with chromatin, 1 × 10^6 ^cells were incubated for 5 min in 1% formaldehyde at 37°C. The cells were harvested, washed with phosphate buffered saline (PBS), resuspended in lysis buffer and 200-1000 bp fragments of DNA from chromatin were prepared as recommended by the manufacturer. One hundredth of the resultant solution was used as an internal control. The remainder was immunoprecipitated for 16 hours at 4°C using anti-acetylated histone H3 and anti-acetylated histone H4 antibodies (Millipore, Billerica, MA). The precipitated immune complexes were recovered using protein A-agarose, and then purified using QIAamp DNA mini kit (QIAGEN). Samples were analyzed by qPCR to determine a ratio of histone acetylation at the *IL-13Rα2 *promoter site using propriety primers Hs04516601_cn for IL-13Rα2 gene and RNase P/TERT reference copy number primers after following the manufacturer's instructions (Applied Biosystems, Foster City, CA).

### Bisulfite-PCR and sequencing

Bisulfite sequencing was performed using CpGenome Fast DNA Modification Kit (Millipore, Billerica, MA). Briefly, 1 μg of genome DNA was incubated for 16 hours at 50°C with sodium bisulfite solution. The modified DNA was purified by DNA binding column. The promoter region of *IL-13Rα2 *gene was amplified by PCR using specific primer pairs, FW: 5'-TTGGGGAGAAAGAGAGATTTG-3', and BW: 5'-CAAACTTACCCCACCCAAAA-3'. The PCR products were cloned into pCR2.1 vector using a TOPO-cloning KIT (Invitrogen, Carlsbad, CA) and sequenced using an ABI377 automated sequencer. At least 10 clones were sequenced for each cell line.

### AP-1 activation assay

Nuclear extracts from cell lines were collected using the Transfactor Extract Kit (Active Motif, Carlsbad, CA) and tested for DNA binding activity using the AP-1 family TransAM Kit (Active Motif) according to the manufacturer's instructions [28].

### Immunohistochemistry (IHC) and Immunocytochemistry (ICC)

Expression of human and mouse IL-13Rα2 protein in pancreatic cancer cell lines and mouse organs was observed by indirect immunofluorescence-immunostaining as described previously [28,30] using anti-mouse monoclonal and anti-human IL-13Rα2 polyclonal antibodies (R&D, Minneapolis, MN). Tissue samples were fixed in 10% formalin solution for IHC and human cells were fixed by 4% paraformaldehyde (PFA) for ICC. The nucleus was counterstained by DAPI.

### *IL-13Rα2 *gene knockdown by RNA interference

Retrovirus-mediated RNA interference was performed using the pSuper RNAi system (Oligoengine, Seattle, WA) following the manufacturer's instructions as described previously [16,28].

### Protein synthesis inhibition assay

*In vitro *cytotoxic activity of IL-13 cytotoxin (IL-13-PE) was measured by the inhibition of protein synthesis as described earlier [11]. All assays were performed in quadruplicate and data are shown as mean ± SD.

### Tumor xenograft studies

Panc-1 and ASPC-1 cells (2 × 10^6^) were injected s.c. in the left flank of female athymic nude mice. From day 4 after tumor implantation, 5 mg/kg TSA was subcutaneously (s.c.) injected every alternative days or 25 mg/kg SAHA were intraperitoneally (i.p.) injected daily for 14 days. From day 5, 50 or 100 μg/kg IL-13-PE or PBS/0.2% human serum albumin (vehicle) were intratumorally (i.t.) injected daily for 14 days. Mice body weight and tumor size was measured every 4-7 days from day 4. Measurement was continued until more than one tumor reached 20 mm in diameter in each group. Their appearances were observed through out the entire experiment for detecting toxic side effects from the treatment. Animal studies were conducted under an approved protocol in accordance with the principles and procedures outlined in *the NIH Guide for the Care and Use of Laboratory Animals*.

### Statistical analysis

The data were analyzed for statistical significance using Student's *t *test for comparison between two groups and ANOVA among more than two groups. All experiments including the animal model were repeated at least twice.

## Results

### *IL-13Rα2 *expression in pancreatic cancer cell lines

**Figure 1:**
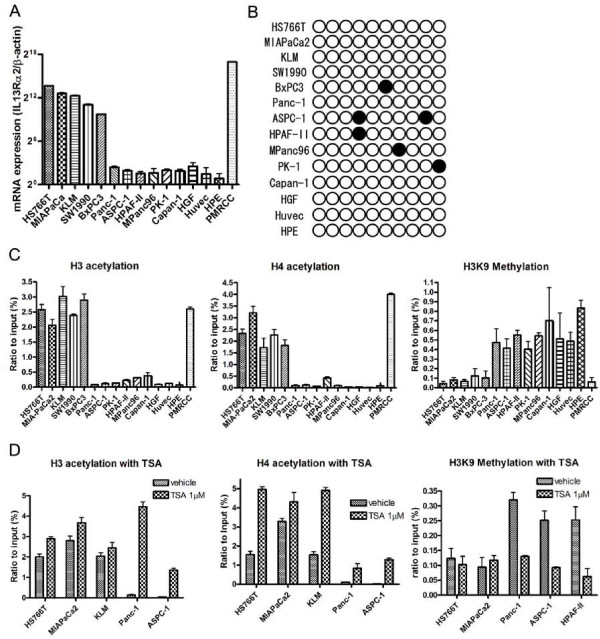
***IL-13Rα2 *expression in pancreatic cancer and normal cell lines and DNA methylation and Histone modification *of IL-13Rα2 *promoter**. A, qRT-PCR for *IL-13Rα2 *expression in pancreatic cancer and normal cell lines was performed. Data shown is ratio of human *IL-13Rα2/β-actin *expression and multiplied by 2^22 ^for convenience. *Bars*, SD of triplicate determinations. B, Bisulfite-sequencing of IL-13Rα2 promoter. Only one CpG site is present within the IL-13Rα2 promoter region. Methylated and unmethylated alleles are shown as solid and open circles, respectively. C, Acetylation and methylation status of histones H3 and H4 in pancreatic cancer and normal cell lines. The region around the *IL-13Rα2 *promoter was amplified by qPCR after ChIP using anti-acetylated histone H3 and H4 antibody and anti-methylated H3K9. Results were standardized by amplification of the *IL-13Rα2 *promoter using DNA before precipitation (Input). D, Acetylation and methylation status of histones H3 and H4 after incubation with TSA. Cells were incubated with 1 μM TSA or vehicle for 24 hours and fixed by 1% PFA. Results were standardized using DNA before precipitation.

Eleven pancreatic cancer cell lines and three types of normal cell lines (fibroblast, umbilical vein endothelial cells and pancreatic ductal epithelial cells) were examined for *IL-13Rα2 *expression. qRT-PCR analysis identified five pancreatic cancer cell lines (HS766T, MIAPaCa2, KLM, SW1990 and BxPC3), which expressed high levels of *IL-13Rα2 *mRNA, and six cell lines (Panc-1, ASPC-1, HPAF-II, Mpanc96, PK-1 and Capan-1) expressed low levels *IL-13Rα2 *mRNA (negative cell line) (Figure 1A). All three normal cell lines showed extremely low levels of *IL-13Rα2 *mRNA. We also examined IL-13Rα2 protein expression in these cell lines by flow-cytometric analysis using monoclonal antibody to IL-13Rα2. These results essentially corroborated the mRNA results (data not shown) [15,31].

### Mutation analysis of IL-13Rα2 cDNA

We investigated whether there were gene sequence changes in the *IL-13Rα2 *gene by performing sequencing of *IL-13Rα2 *cDNA. However, no mutations were detected in any pancreatic cancer cell lines studied (data not shown).

### DNA methylation in *IL-13Rα2 *promoter

We next examined any epigenetic changes in *IL-13Rα2 *gene. Since there is only one CpG site in the *IL-13Rα2 *promoter region, we examined DNA methylation at this site [32]. We picked more than 10 independent clones for analysis. In at least 80% of the clones tested from all cell lines including three normal cell lines, no methylation was detected (Figure 1B). As a control, we also studied DNA methylation of other CpG sites located ~100 bases upstream from the *IL-13Rα2 *promoter region. In contrast to the CpG in the IL-13Rα2 promoter region, the distant CpG site showed methylation in all cell lines (Supplementary Figure 1).

### Regulation of histone acetylation and methylation in *IL-13Rα2 *promoter region

We also examined histone acetylation of the *IL-13Rα2 *promoter region using a chromatin-immunoprecipitation technique (ChIP). In all IL-13Rα2-positive pancreatic cell lines, histone H3 was highly acetylated compared to IL-13Rα2-negative and normal cell lines (Figure 1C). Similar acetylation results were observed for histone H4. In sharp contrast, the methylation status at the H3K9 site, which is a site for transcriptional repression, was high in IL-13Rα2-negative cell lines compared to IL-13Rα2-positive cell lines (Figure 1C).

Next, we examined the effect of histone acetylation inhibition by HDAC inhibitors on IL-13Rα2 expression. When pancreatic cancer lines expressing undetectable levels of *IL-13Rα2 *were treated with TSA, histone H3 and H4 acetylation was dramatically increased. TSA also increased acetylation in pancreatic cancer cells expressing high levels of IL-13Rα2 but this increase was less dramatic (Figure 1D). In contrast, TSA caused a significant decrease in H3K9 methylation in pancreatic cancer cells with undetectable levels of IL-13Rα2 expression but no change in high IL-13Rα2 expressing cell lines (Figure 1D).

### Histone deacetylation inhibition increases *IL-13Rα2 *expression in pancreatic cancer cell lines

**Figure 2:**
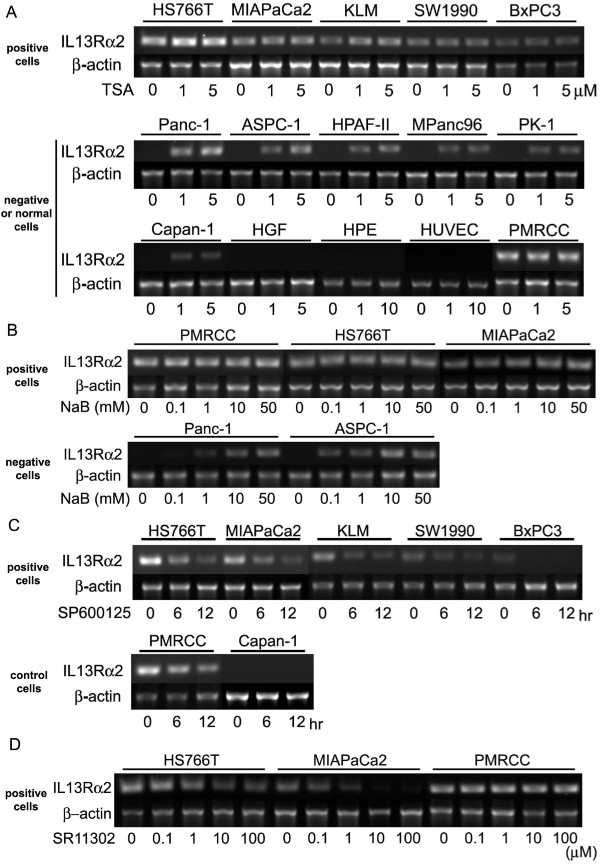
**Regulation of *IL-13Rα2 *expression by HDAC and AP-1 inhibitors**. A, Conventional RT-PCR of *IL-13Rα2 *mRNA after incubation with TSA. Cells were incubated with 1 or 5 μM TSA for 24 hours and total RNA was extracted. PM-RCC cells were used as a positive control. β-actin is shown as a reference gene. B, Conventional RT-PCR of *IL-13Rα2 *after incubation with NaB. Cells were incubated with 0 - 50 mM NaB for 24 hours and total RNA extracted. C, Conventional RT-PCR of *IL-13Rα2 *gene after incubation with SP600125. Cells were incubated with 10 μM SP600125 for 6 or 12 hours and total RNA extracted. D, Conventional RT-PCR of *IL-13Rα2 *after incubation with AP-1 inhibitor, SR11302. Cells were incubated with 0 - 100 μM SR11302 for 12 hours and total RNA extracted.

As the relationship between histone acetylation and *IL-13Rα2 *expression levels was observed, we tested whether HDAC inhibitors can modulate *IL-13Rα2 *expression in pancreatic cancer cell lines. Interestingly, similar to histone acetylation, TSA treatment resulted in increased *IL-13Rα2 *mRNA expression in pancreatic cancer cell lines that normally have undetectable levels of IL-13Rα2 expression, while no changes were seen in cells expressing high levels of *IL-13Rα2 *mRNA or normal cell lines (Figure 2A). Similar results were obtained with another HDAC inhibitor, sodium butyrate (NaB) (Figure 2B).

### Role of AP-1 transcription factor activity in IL-13Rα2 regulation in pancreatic cancer cell lines

To determine the mechanism of the differential effect of HDAC inhibition in cells expressing undetectable levels of IL-13Rα2, we examined whether the transcription factor (AP-1) is activated in these cell lines as reported by Wu et al. [32]. We found that pancreatic cancer cell lines that highly express *IL-13Rα2 *(HS766T, MIAPaCa2, and KLM), and those which express undetectable levels (Panc-1 and ASPC-1), both show high c-jun activity (Supplementary Figure 2A). In contrast, normal cell lines showed low c-jun activity. We did not observe any significant differences in c-Fos activity, another AP-1 member (Supplementary Figure 2B) between cancer and normal cell lines.

**Figure 3:**
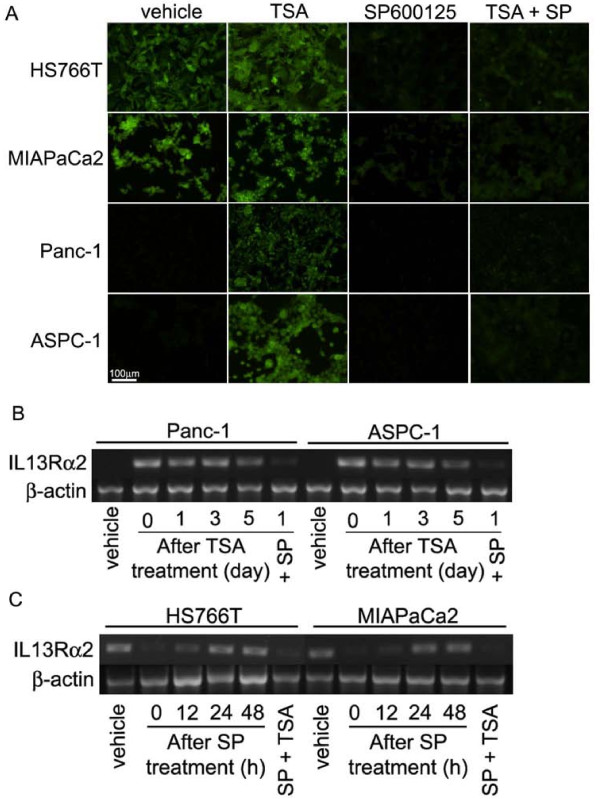
**Modulation of IL-13Rα2 protein by HDAC and AP-1 inhibitors and stability of *IL-13Rα2 *expression**. A, ICC of IL-13Rα2 after incubation with TSA and SP600125 is shown. Cells were incubated with 1 μM TSA and/or 10 μM SP600125 for 24 hours and fixed by 4% PFA. IL-13Rα2 was visualized by Alexa488. Recovery of IL-13Rα2 expression after incubation with TSA (B) and SP600125 (C). Cells were incubated with 1 μM TSA or SP600125 for 24 hours or 12 hours, respectively and then inhibitors were removed by replacing with new medium without TSA for 1-5 days or SP600125 for 12-48 hours. *IL-13Rα2 *gene expression was determined by conventional RT-PCR.

Interestingly, when high IL-13Rα2-expressing cells were treated with the c-jun N-terminal kinase inhibitor, SP600125, *IL-13Rα2 *expression decreased (Figure 2C), whereas SP600125 had no effect on cells expressing undetectable levels of *IL-13Rα2*. Another pan-AP-1 inhibitor, SR11302, also decreased *IL-13Rα2 *expression in IL-13Rα2 expressing cell lines in a concentration-dependent manner (Figure 2D). The effects of TSA and SP600125 on IL-13Rα2 protein expression in pancreatic cancer cells were also analyzed by IHC. IL-13Rα2 protein levels were also found to increase in the presence of TSA and decrease in the presence of SP600125. In addition, SP600125 prevented the increase of IL-13Rα2 protein by TSA (Figure 3A).

### Stability of upregulated *IL-13Rα2 *expression by HDAC inhibitor

We examined the stability of upregulated *IL-13Rα2 *expression in IL-13Rα2-expressing and negative pancreatic cancer cell lines when treated with HDAC inhibitor. After treatment with TSA and SP600125 for 24 hours, the drugs were removed and cell culture was continued. *IL-13Rα2 *expression was still elevated 3 days after TSA removal in *IL-13Rα2 *undetectable cell lines (Figure 3B). In contrast, in IL-13Rα2 positive cell lines, *IL-13Rα2 *expression returned to pre-treatment levels within 24 hours following SP600125 removal (Figure 3C).

### HDAC inhibition increases IL-13 induced matrix metalloproteinases via IL-13Rα2 upregulation

**Figure 4:**
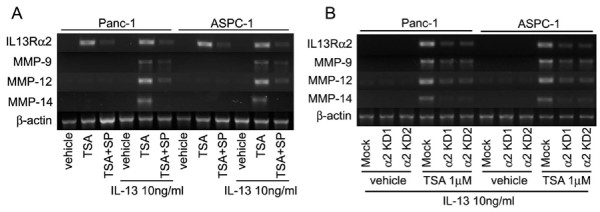
**HDAC inhibitor inhibits MMPs expression activated by IL-13 through induction of IL-13Rα2**. A, Conventional RT-PCR for expression of *MMPs *was performed after cells were incubated with 1 μM TSA and/or 10 μM SP600125 for 24 hours. Twenty-two hours prior to harvesting cells, IL-13 was added to the cultured medium and total RNA extracted. β-actin is shown as a reference gene. B, *MMPs *expression in IL-13Rα2 knock-down (α2KD) cells incubated with TSA. Mock and α2KD cells were treated with TSA and IL-13 same as in panel B.

As we have shown that IL-13 can upregulate Matrix metalloproteinases (MMPs) expression in IL-13Rα2 expressing pancreatic cancer cell lines [28], we investigated the impact of IL-13Rα2 upregulation by HDAC inhibitors by examining IL-13 induced MMPs expression. TSA treatment increased mRNA expression for *MMPs *through upregulation of *IL-13Rα2 *after treatment with IL-13 in two IL-13Rα2 negative cell lines (Figure 4A). Interestingly, when IL-13 signaling was blocked by an inhibitor of the AP-1 pathway (SP600125), it prevented the increase in MMPs expression by TSA. Thus, *MMPs *expression showed a positive correlation with *IL-13Rα2 *expression in IL-13 treated cells.

To confirm whether TSA increased *MMPs *expression as a result of IL-13Rα2 induction, we conducted a knock-down of the *IL-13Rα2 *gene using two different sequences of siRNA in Panc-1 and ASPC-1 cell lines. *MMPs *expression was suppressed in *IL-13Rα2 *knock-down cells treated with TSA (Figure 4B).

### HDAC inhibition increases the anti-cancer effect of IL-13-PE targeting IL-13Rα2 *in vitro *and *in vivo*

**Figure 5:**
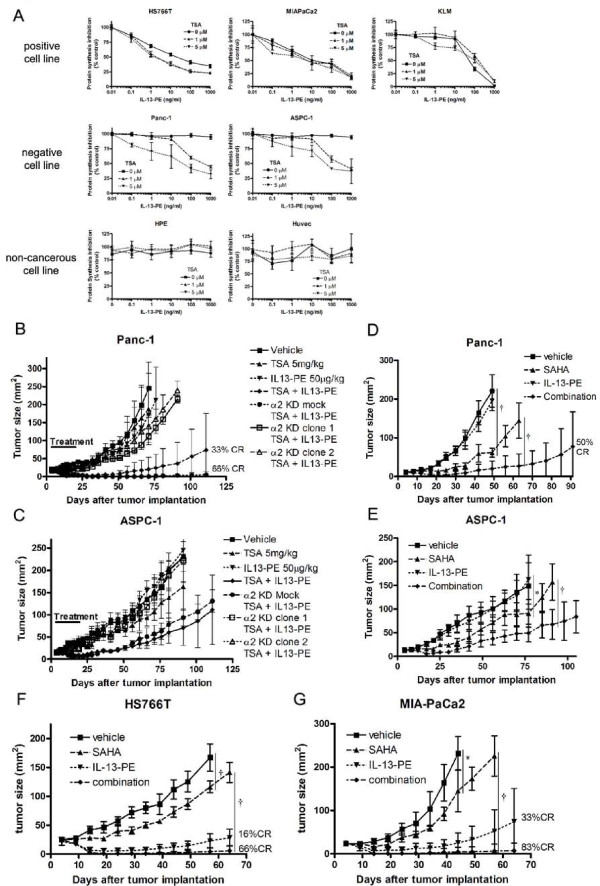
**HDAC inhibitors induce anti tumor effect of IL-13Rα2 targeted immmunotoxin IL13-PE in IL-13Rα2-negative pancreatic cancer cell lines**. A, Cytotoxicity assay was performed in IL-13Rα2-negative and -positive pancreatic cancer and normal cell lines. Cells were pre-treated with 0 - 5 μM TSA for 24 hours and then treated with 0 - 1000 ng/ml IL-13-PE for 20 hours in leucine-free medium. Protein synthesis was evaluated by H^3^-leucine incorporation. Percentage cytotoxicity was calculated with no treatment control as 100%. B and C, Regression of IL-13Rα2-negative pancreatic tumors (Panc-1 and ASPC-1) treated with 5 mg/kg TSA and/or 100 μg/kg IL-13-PE as described in methods. Mock combination means tumors were mock transected with control vector and treated with HDAC inhibitors and IL-13-PE in vivo. D and E, Regression of IL-13Rα2-negative pancreatic tumors treated with SAHA and/or IL-13-PE. Mice were treated daily with i.p. injection of SAHA (25 mg/kg) from day 4 after tumor implantation for two weeks followed by i.t. injection of IL-13-PE (100 μg/kg) from day 5 for two weeks. F and G, Regression of IL-13Rα2-posotive pancreatic tumors (HS766T and MIA-PaCa2) treated with SAHA and/or IL-13-PE. The schedule of treatment was similar as in panel D and E. Statistical significances are shown by *: P < 0.05, †: P < 0.001.

As HDAC inhibition increased IL-13Rα2 expression in IL-13Rα2-negative but not in normal cell lines, we examined whether HDAC inhibition enhanced the anti-cancer effect of IL-13-PE in IL-13Rα2-negative pancreatic cancer cell lines. The anti-cancer effect of IL-13-PE was evaluated using a protein synthesis inhibition assay *in vitro *(Figure 5A). IL-13-PE inhibited protein synthesis in IL-13Rα2-positive cancer cells (IC_50 _between 10 and 50 ng/ml) without TSA, but not in IL-13Rα2-negative cancer cells nor normal cells (IC_50 _> 1000 ng/ml). TSA treatment enhanced the cytotoxicity of IL-13-PE in IL-13Rα2-negative cancer cells (IC_50 _40-50 ng/ml with 5 μM TSA), but not in normal cells (IC_50 _> 1000 ng/ml with 5 μM TSA).

We next examined the enhancement of the anti-cancer effect of IL-13-PE by HDAC inhibition in xenograft mouse models of human cancer. IL-13Rα2-negative pancreatic cancer cell lines (Panc-1 and ASPC-1) were implanted in the flanks of immunodeficient mice and treated with two different HDAC inhibitors, TSA and SAHA followed by IL-13-PE immunotoxin. Neither TSA nor IL-13-PE alone affected the tumor growth, but when combined, a dramatic inhibition of tumor growth was observed (Figure 5B and 5C). In contrast, when *IL-13Rα2 *was knocked-down prior to TSA therapy, the anti-tumor effect of combination of TSA and IL-13-PE was completely eliminated compared to mock vector transfected tumors, which showed dramatic tumor response (Figure 5B).

A second HDAC inhibitor, SAHA, itself showed some anti-cancer effect in two tumor models (Figure 5D and 5E). However, when mice were treated with SAHA followed by IL-13-PE, a significant decrease in tumor size was observed. In addition, 50% of mice showed complete elimination of their tumors in combination group.

Next, we evaluated anti-cancer effect of combination of SAHA and IL-13-PE in IL-13Rα2-positive pancreatic cancer model (HS766T and MIA-PaCa2). We observed that IL-13-PE could significantly decrease tumor size in both IL-13Rα2-positive tumors (Figure 5F and 5G). But when combined with SAHA, IL-13-PE not only decreased tumor size but also completely eliminated tumors in 66 to 83% of mice. These data suggest that SAHA can enhance anti-cancer effect of IL-13-PE even in IL-13Rα2-positive pancreatic cancers.

We monitored the body weight of mice and their general condition throughout the experimental period and detected no adverse effects caused by the treatment (data not shown). In addition, we observed no organ toxicity in vital organs such as the liver, brain, lung, kidney, pancreas and spleen of IL-13-PE and HDAC inhibitor-treated mice evaluated by histological examination (Supplementary Figure 3)

### HDAC inhibitor significantly increased IL-13Rα2 in the pancreatic tumors implanted in the mice but not in mice organs

**Figure 6:**
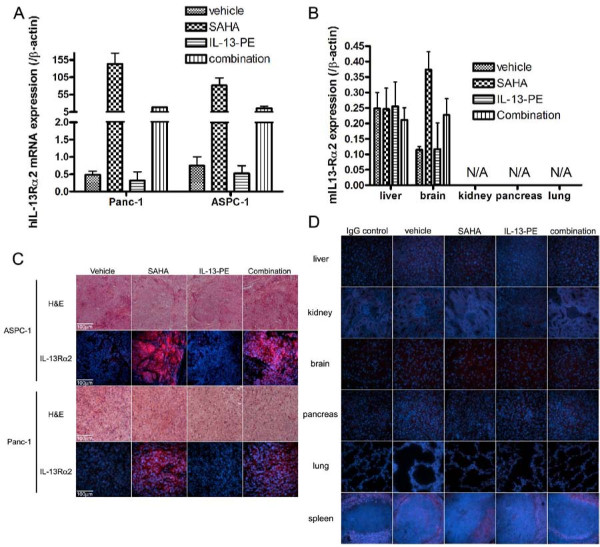
**IL-13Rα2 expression is upregulated in pancreatic tumors but not in organs of mice after treatment with HDAC inhibitor, SAHA**. A, qRT-PCR of human *IL-13Rα2 *in implanted pancreatic tumors after SAHA and IL-13-PE treatment. Tumors were harvested next day after IL-13-PE treatment and total RNA extracted. Data shown is ratio of human *IL-13Rα2/β-actin *expression and multiplied by 1000 for convenience. *Bars*, SD of triplicate determinations. B, qRT-PCR of mouse IL-13Rα2 in mice organs after SAHA and IL-13-PE treatment. Tissues were harvested at the same time point as in panel A and total RNA extracted. Data shown is ratio of mouse *IL-13Rα2/β-actin *expression and multiplied by 100 for convenience. C, IHC of human IL-13Rα2 in implanted pancreatic tumors after SAHA and IL-13-PE treatment. D, IHC of mouse IL-13Rα2 in mice organs after SAHA and IL-13-PE treatment. Liver, brain, kidney, pancreas, lung and spleen were fixed for immunostaining of mouse IL-13Rα2 as visualized by Alexa555. Nucleus was counterstained by DAPI.

After SAHA and IL-13-PE treatment, implanted tumors and mice organs (liver, brain, pancreas, kidney, spleen and lung) were harvested and IL-13Rα2 expression was examined at mRNA and protein levels. Human *IL-13Rα2 *mRNA was significantly increased in tumors in both SAHA treated mice (Figure 6A) and TSA treated mice (Supplementary Figure 4). IL-13-PE treatment had no effect by itself but in combination with SAHA, a significant decrease in *IL-13Rα2 *expression was observed. In contrast, none of the organs except brain showed a modest increase in mouse *IL-13Rα2 *mRNA expression (Figure 6B).

We also examined IL-13Rα2 protein expression by IHC. Similar to mRNA results, human IL-13Rα2 was dramatically increased in tumors from SAHA treated mice and when combined with IL-13-PE, a decrease in IL-13Rα2 expression was observed (Figure 6C). In normal tissues, mouse IL-13Rα2 was not detected or levels were below the detection limit of the assay in all organs examined (Figure 6D).

## Discussion

We demonstrate for the first time that *IL-13Rα2*, a tumor antigen, is highly susceptible to epigenetic modulation in pancreatic cancer cell lines. Interestingly, DNA methylation and histone acetylation were differentially regulated in cells overexpressing or not overexpressing IL-13Rα2. Histones (H3 and H4) were highly acetylated at the promoter region of *IL-13Rα2 *in IL-13Rα2-positive pancreatic cancer cell lines, but not in IL-13Rα2-negative cell lines. In contrast, histones in IL-13Rα2-negative pancreatic cell lines and normal cell lines were highly methylated, but not in IL-13Rα2 positive cell lines. The reason for the differential histone acetylation and methylation is not known but appears to correlate with IL-13Rα2 expression and may be responsible for variability of IL-13Rα2 expression in cancer cells.

The role of histone acetylation was explored further using histone deacetylase (HDAC) inhibitors. Interestingly, in the presence of HDAC inhibitors (TSA and NaB), IL-13Rα2 expression was significantly induced in IL-13Rα2-negative cell lines whose histones were not acetylated compared to IL-13Rα2-positive cell lines in which histones were acetylated. The mechanism of differential IL-13Rα2 regulation was examined. IL-13 signals through IL-13Rα2 via the AP-1 pathway and inactivation of this pathway by JNK and AP-1 inhibition suppressed IL-13Rα2 expression in IL-13Rα2-positive cell lines. Additionally, inactivation of the AP-1 pathway also suppressed induction of IL-13Rα2 by HDAC inhibitors in IL-13Rα2-negative cell lines. In accordance, Wu et al. have reported the importance of c-jun, which is a member of AP-1 transcription factor, in IL-13Rα2 expression [32]. These observations indicate a strong correlation between transcription factor and histone acetylation in the IL-13Rα2 at the promoter region.

The significance of IL-13Rα2 upregulation by HDAC inhibitors was examined. As expected, IL-13 induced STAT6 phosphorylation in IL-13Rα2-negative pancreatic cancer cell lines (Supplementary Figure 5). Interestingly, TSA increased IL-13Rα2 expression, but suppressed STAT6 phosphorylation induced by IL-13 treatment. The suppression of STAT6 phosphorylation by TSA was inhibited by IL-13Rα2 RNAi indicating that IL-13Rα2 is directly involved in this counter-regulation (data not shown). Similarly, as expected, IL-13 did not induce MMPs expression in IL-13Rα2-negative pancreatic cancer cell lines [28]. However, when cells were treated with TSA, IL-13 could increase MMP-9, 12 and 14 mRNA as IL-13Rα2 expression was upregulated. In contrast, MMPs were not induced by TSA when IL-13Rα2 was knocked-down by RNAi or IL-13 signaling was inhibited by JNK inhibitor.

We took advantage of upregulation of IL-13Rα2 in pancreatic cancer cell lines and hypothesized that HDAC inhibitors may enhance the sensitivity of IL-13 receptor-targeted immunotoxin, IL-13-PE, in pancreatic cancers. We have previously demonstrated that IL-13-PE is a powerful anti-cancer agent, causing regression of IL-13Rα2-positive human tumors derived from variety of human cancers including pancreatic cancer [15,16]. However, for efficacy, these tumors must express high levels of IL-13Rα2. Since cancer is a heterogeneous disease, drug-induced upregulation of IL-13Rα2 could be used in cancers expressing even low levels of IL-13 α2 to enhance the intensity of the immunotoxin anti-cancer response. Indeed, we demonstrate that pre-treatment of tumor cell lines *in vitro *with TSA enhanced their sensitivity to IL-13-PE and made IL-13Rα2-negative cell lines extremely sensitive to IL-13-PE. In contrast, TSA treatment did not sensitize normal epithelial cell lines, thus providing a therapeutic advantage of targeting tumors but not normal tissues. Consequently, the use of HDAC inhibitors may open a new avenue of treating pancreatic cancer when combined with IL-13-PE. It is possible that HDAC inhibitors may also sensitize tumors to other immunotoxins targeting different antigens or cell surface receptors.

The reason why normal epithelial cells are not sensitized to IL-13-PE by TSA is not clear. Epithelial cells exhibit a similar histone modification pattern to IL-13Rα2-negative pancreatic cancer cell lines but, IL-13Rα2 is not upregulated in normal epithelial cells by HDAC inhibitors. This may be because normal cell lines show no c-jun activity, while IL-13Rα2-negative pancreatic cancer cell lines show a 2-6 fold increase in c-jun activity indicating that TSA induction of high levels of IL-13Rα2 is dependent on the AP-1/c-jun pathway.

We also demonstrate that HDAC inhibitors when combined with IL-13-PE cause more dramatic tumor responses than those caused by either agent alone in two pancreatic cancer models. Pancreatic cancers in situ were not sensitive to IL-13-PE as they do not naturally express IL-13Rα2 and TSA or SAHA alone showed only modest to moderate anti-tumor effects. However, when TSA or SAHA were combined with IL13-PE a dramatic inhibition of tumor growth was observed. In agreement with our observations, HDAC inhibition has been reported in combination therapies for other types of cancer. Combination therapy of SAHA and retinoic acid has been examined for resistant acute promyelocytic leukemia in which SAHA enhanced the anti-cancer effect of retinoic acid [33]. Another HDAC inhibitor, LAQ824, is reported to be effective in combination with adoptive T-cell transfer therapy against mouse model of melanoma [34]. These authors hypothesized that LAQ824 increases the tumor-associated antigen expression enhancing the anti-tumor effectiveness of T cell therapy.

It is important to note that while HDAC inhibition enhanced the remarkable anti-cancer effects of IL-13-PE in pancreatic cancer models *in vivo *by upregulating IL-13Rα2 in the tumors, no significant upregulation of IL-13Rα2 expression was observed in any vital organs. In addition, no detectable histological changes were observed in any vital organs. Although IL-13-PE was injected locally, our findings confirm that this novel combination therapeutic approach is safe. Future studies will examine systemic administration of IL-13-PE in combination with HDAC inhibitors in syngenic animal tumor models. Taken together, our results provide support for testing this novel combination in the clinic for the therapy of human cancer including pancreatic cancer for which no therapeutic options are currently available.

## Abbreviations

IL-13Rα2: interleukin 13 receptor alpha 2; IL-13-PE: interleukin 13 *pseudomonas *exotoxin.

## Competing interests

The authors declare that they have no competing interests.

## Authors' contributions

Conceived and designed the experiments: TF, BHJ, RKP. Performed the experiments: TF. Analyzed the data: TF. Wrote the paper: TF, BHJ, RKP.

All authors have read and approved the final manuscript.

## Supplementary Material

Additional file 1 **Figure S1: DNA methylation status of upstream sequences from *IL-13Rα2 *promoter site**. DNA methylation status was examined by bisulfite-sequencing at the CpG site located about 100 bases upstream from *IL-13Rα2 *promoter region. Methylated and unmethylated alleles are shown as solid and open circles, respectively. Click here for file

Additional file 2 **Figure S2: AP-1 transcription factor activity in pancreatic cancer cell lines**. c-jun (A) and c-Fos (B) activity in pancreatic cancer and normal cell lines. Protein samples were extracted from nuclear fraction. AP-1 activity was measured by ELISA. Click here for file

Additional file 3 **Figure S3: Histological finding of vital organs in SAHA and IL-13-PE treated mice**. Tissue specimens were obtained from mice liver, kidney, spleen, pancreas, brain and lung in each group of SAHA and IL-13-PE treated experiment (day 19) for hematoxylin and eosin staining. Click here for file

Additional file 4 **Figure S4: *IL-13Rα2 *expression is upregulated in pancreatic tumors after treatment with TSA**. qRT-PCR of human *IL-13Rα2 *in implanted human pancreatic tumors, Panc-1 (A) and ASPC-1 (B) after TSA and IL-13-PE treatment. Tumors were harvested next day after IL-13-PE treatment ended and total RNA was extracted. Data shown is ratio of human *IL-13Rα2/β-actin *expression. *Bars*, SD of triplicate determinations. Click here for file

Additional file 5 **Figure S5: HDAC inhibitor inhibits IL-13 induced STAT6 activation through induction of IL-13Rα2**. Western blotting of phospho- and total STAT6 after incubation of cells with TSA and/or SP600125. Cells were incubated with 1 μM TSA and/or 10 μM SP600125 for 24 hours. Fifteen minutes before harvest, IL-13 was added to the culture medium. Protein samples were prepared from nuclear compartment and separated by electrophoresis. Click here for file
