# Supplementary figures and images for: Histone modification enhances the effectiveness of IL-13 receptor targeted immunotoxin in murine models of human pancreatic cancer
Source: J Transl Med. 2011 Apr 8;9:37. doi: 10.1186/1479-5876-9-37 (PMC3096924; doi:10.1186/1479-5876-9-37)

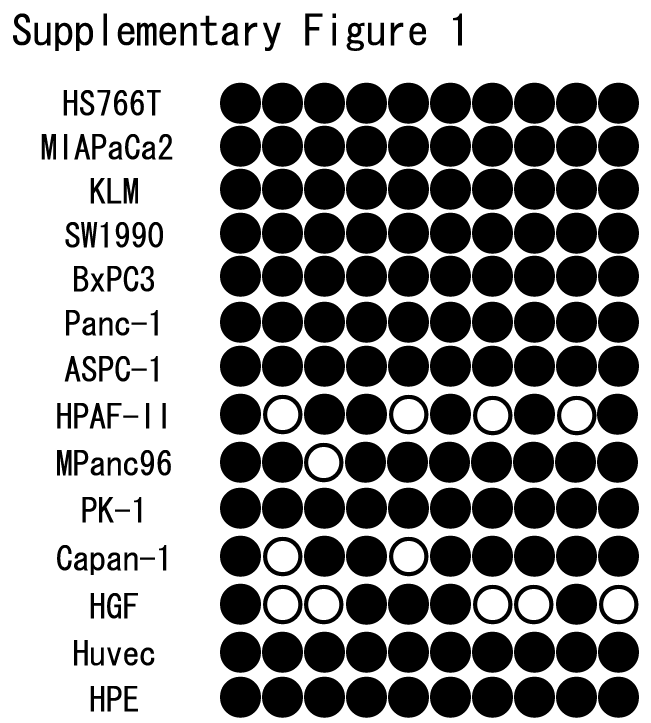

Supplement: Additional file 1 — Figure S1: DNA methylation status of upstream sequences from IL-13Rα2 promoter site. DNA methylation status was examined by bisulfite-sequencing at the CpG site located about 100 bases upstream from IL-13Rα2 promoter region. Methylated and unmethylated alleles are shown as solid and open circles, respectively. [file 1479-5876-9-37-S1.TIFF]

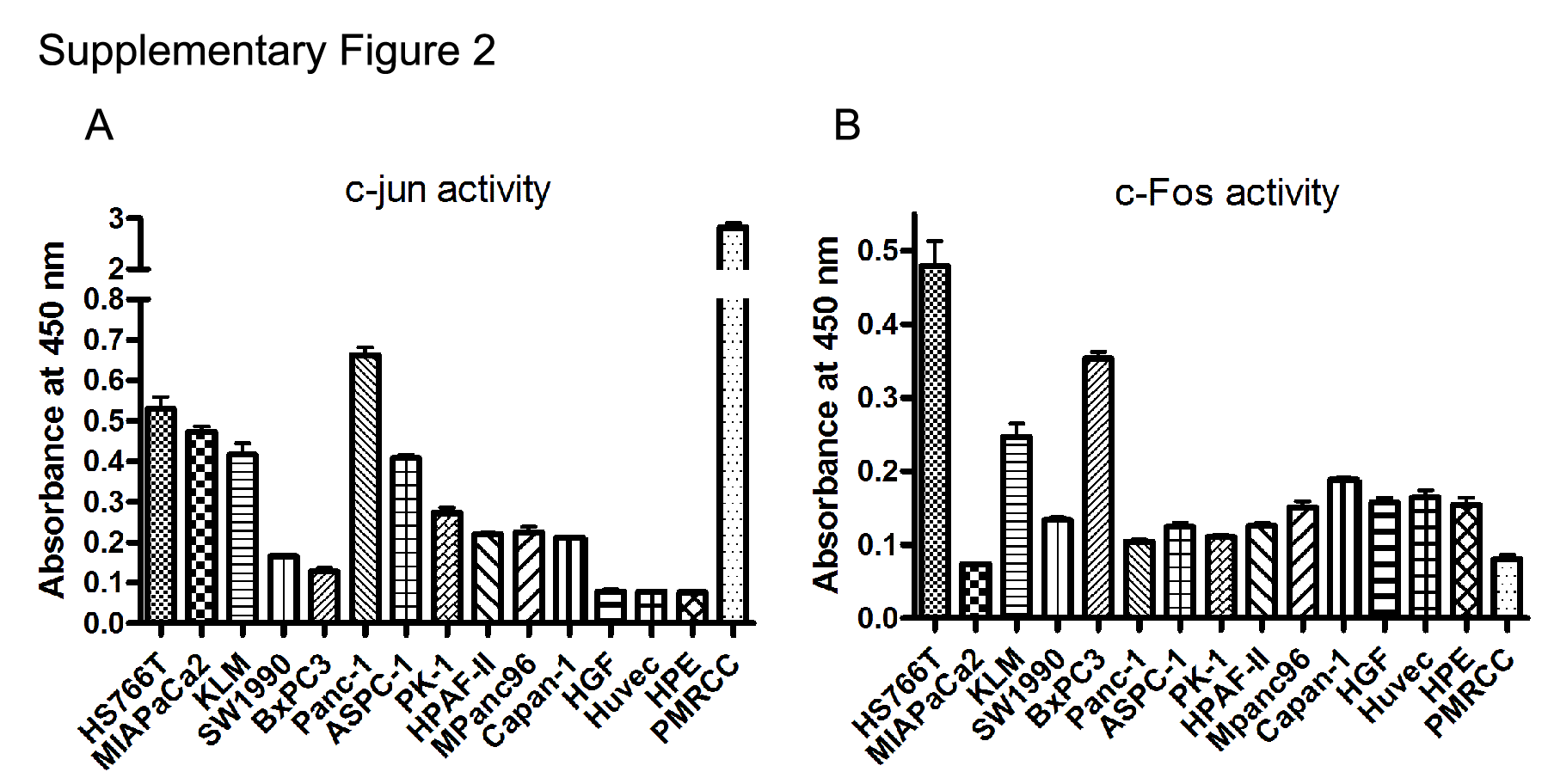

Supplement: Additional file 2 — Figure S2: AP-1 transcription factor activity in pancreatic cancer cell lines. c-jun (A) and c-Fos (B) activity in pancreatic cancer and normal cell lines. Protein samples were extracted from nuclear fraction. AP-1 activity was measured by ELISA. [file 1479-5876-9-37-S2.TIFF]

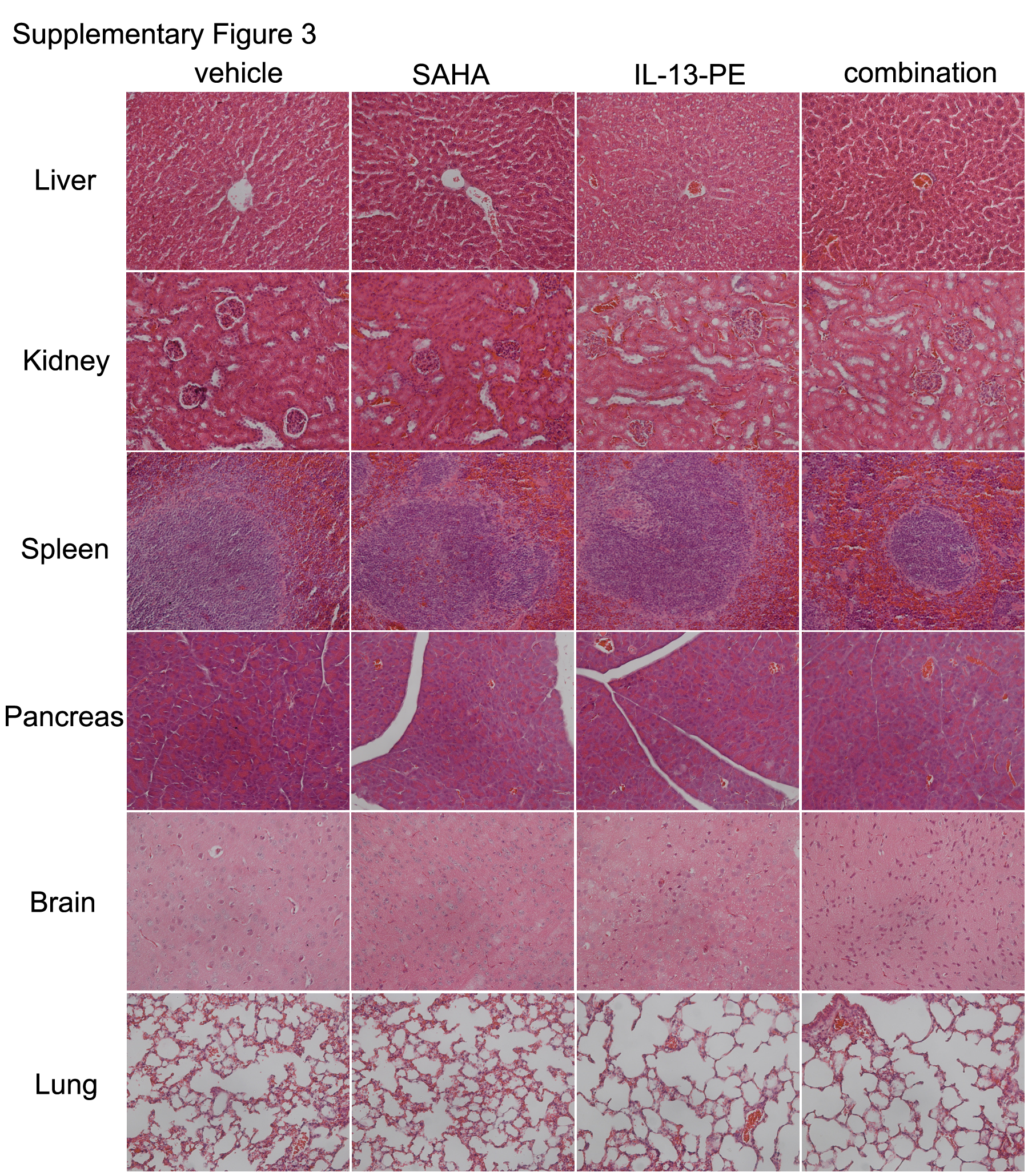

Supplement: Additional file 3 — Figure S3: Histological finding of vital organs in SAHA and IL-13-PE treated mice. Tissue specimens were obtained from mice liver, kidney, spleen, pancreas, brain and lung in each group of SAHA and IL-13-PE treated experiment (day 19) for hematoxylin and eosin staining. [file 1479-5876-9-37-S3.TIFF]

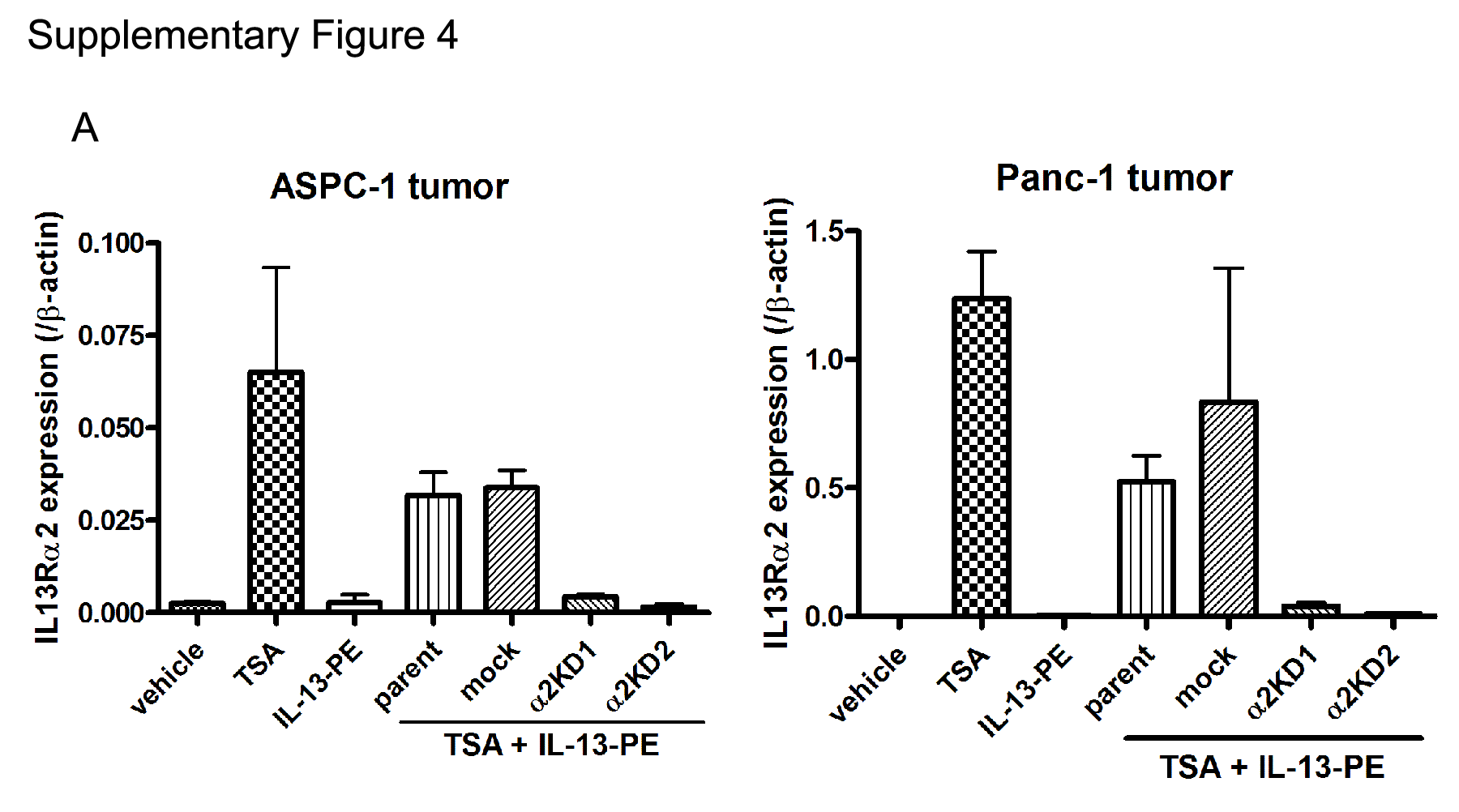

Supplement: Additional file 4 — Figure S4: IL-13Rα2 expression is upregulated in pancreatic tumors after treatment with TSA. qRT-PCR of human IL-13Rα2 in implanted human pancreatic tumors, Panc-1 (A) and ASPC-1 (B) after TSA and IL-13-PE treatment. Tumors were harvested next day after IL-13-PE treatment ended and total RNA was extracted. Data shown is ratio of human IL-13Rα2/β-actin expression. Bars, SD of triplicate determinations. [file 1479-5876-9-37-S4.TIFF]

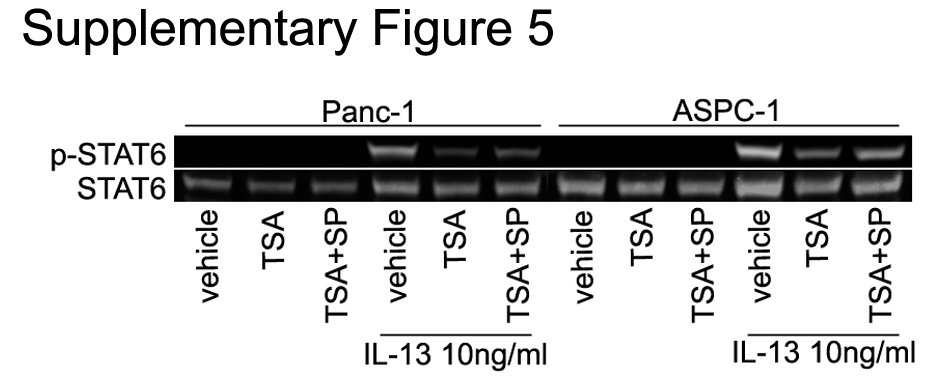

Supplement: Additional file 5 — Figure S5: HDAC inhibitor inhibits IL-13 induced STAT6 activation through induction of IL-13Rα2. Western blotting of phospho- and total STAT6 after incubation of cells with TSA and/or SP600125. Cells were incubated with 1 μM TSA and/or 10 μM SP600125 for 24 hours. Fifteen minutes before harvest, IL-13 was added to the culture medium. Protein samples were prepared from nuclear compartment and separated by electrophoresis. [file 1479-5876-9-37-S5.TIFF]
